# Distribution of the Main *Apis mellifera* Mitochondrial DNA Lineages in Italy Assessed Using an Environmental DNA Approach

**DOI:** 10.3390/insects12070620

**Published:** 2021-07-08

**Authors:** Valerio Joe Utzeri, Anisa Ribani, Valeria Taurisano, Carles Hernández i Banqué, Luca Fontanesi

**Affiliations:** Department of Agricultural and Food Sciences, University of Bologna, Viale Giuseppe Fanin 46, 40127 Bologna, Italy; valeriojoe.utzeri2@unibo.it (V.J.U.); anisa.ribani2@unibo.it (A.R.); valeria.taurisano2@unibo.it (V.T.); carles.hernandez.banque@gmail.com (C.H.i.B.)

**Keywords:** conservation genetics, eDNA, honey, Italian peninsula, latitude, mitotype, mtDNA, population genetics, Sicily

## Abstract

**Simple Summary:**

The conservation of the genetic diversity of the native honey bee subspecies is a hot topic in many European countries. Mitochondrial DNA (mtDNA) analyses can provide some information that is useful to monitor the genetic integrity of *Apis mellifera* populations. A preliminary distribution of the main honey bee mitotypes in Italy was obtained more than 20 years ago. In this study we obtained an updated and more detailed distribution map of the main groups of honey bee mitotypes using an unconventional method that exploits the information derived from the environmental DNA contained in the honey. The results were quite different from the picture taken two decades ago. The African mtDNA lineage was spread all over Italy and not only in Sicily, where it is mainly attached to the *A. m. siciliana* subspecies, and where it was identified in the previous investigation. A reduction in the frequency of the M lineage was also evident, and, on the other hand, a counterbalanced increase in the C mitotypes was observed in regions all over. The obtained results provided an updated distribution map of the A, C and M groups of mitotypes in Italy, which could be a starting point to design appropriate conservation programs for native honey bee subspecies.

**Abstract:**

Growing interest has been emerging on the need to monitor the genetic integrity of the European *Apis mellifera* subspecies that could be threatened by the human-mediated dispersion of non-native populations and lines. Mitochondrial DNA (mtDNA) lineages can provide useful information for this purpose. In this study, we took advantage of the environmental DNA (eDNA) contained in the honey, which can be analyzed to detect the main groups of mitotypes of the honey bees that produced it. In this study, we applied this eDNA to produce a distribution map all over the Italian peninsula and the two major islands (Sicily and Sardinia) of the following three honey bee mtDNA lineages: A, C and M. A total of 607 georeferenced honey samples, produced in all Italian regions, was analyzed to detect these lineages. The A lineage was widespread in Sicily, as expected, considering that *A. m. siciliana* carries the African lineage. Surprisingly, this lineage was also reported in about 14% of all other samples produced in almost all continental regions, and in Sardinia. The applied method obtained an updated distribution map of honey bee mtDNA lineages that could be useful to design policies for the conservation of Italian honey bee genetic resources.

## 1. Introduction

The genetic integrity of honey bee (*Apis mellifera*) populations and subspecies, which might be well adapted to local environments, is a matter of growing attention in several European countries [1,2,3,4,5,6,7]. The maintenance of locally adapted genetic resources is considered critical for the long-term survival and sustainability of beekeeping activities, and the related ecosystem services of pollination in the agroecological environments and agricultural production systems [3,8,9].

The erosion of the genetic diversity among honey bee populations is caused by the combined action of different factors [10]. On the one hand, beekeeping activities, including the transhumance of colonies and extensive trading of queen bees, may contribute to increasing the admixture and reducing, in turn, the genetic diversity between the populations [11,12,13]. On the other hand, the action of multifactorial elements (including the extensive use of harmful pesticides for the bees, the effect of adverse climate conditions, and the related increased sensitivity to parasites and pathogens), indicated as the main causes of the global decline in the bees, contributes to the erosion of the population size of *A. mellifera*, which incentives the use of non-autochthonous genetic stocks to replace the dead colonies, usually when local breeding programs and queen production cannot satisfy the requests of the local beekeepers [14,15,16]. Transhumance is also needed, in several cases, to counterbalance the effects of climate change on nectar availability [17,18,19,20].

About 30 *A. mellifera* subspecies have been described thus far, starting from morphometrical differences that are summarized by several classical studies in this field [21,22,23,24,25], and then complemented by investigations on the mitochondrial DNA (mtDNA) and nuclear genome variability [26,27,28,29,30,31]. These subspecies have been grouped into five major evolutionary lineages (A, C, M, O and Y), the following three of which are considered to be originally present in different European regions [25]: A, the African lineage, which was mainly spread through Southern Europe (Iberian peninsula, the close Gascony in Southwest France, and several Mediterranean islands, including Sicily); C, widespread in the east of Europe and in the Italian peninsula; M, which generally covers the northern part of Western Eurasia, from the British Isles through most of continental Europe, to the Ural and some areas in Central Asia. These lineages, with their characteristic mitotypes matching in part to morphometric features, include the following several subspecies: *A. m. iberiensis* (in the Iberian peninsula), which has both A and M mitotypes according to its hybrid origin [32,33,34,35,36,37,38]; *A. m. siciliana* (in the Sicily island and close minor islands), which has A mitotypes [39,40,41]; *A. m. ligustica* (in the Italian peninsula), which mainly carries the C1 mitotype, but also the M7 mitotype, supporting its suggested hybrid origin, due to the refugee of the M branch in the Apennine peninsula during the quaternary ice period [40]; *A. m. mellifera* (in Western and North Europe), which carries the M mitotypes [26,27,28,35,42,43]; and *A. m. carnica* (in East-Central Europe), which mainly carries the C2 mitotype [44,45,46].

Due to its geographical position, Italy is a unique case in Europe, as it hosts natural populations of the four latest subspecies indicated above [6,25,40,42,47,48,49,50]. In addition to *A. m. ligustica* and *A. m. siciliana*, *A. m. mellifera* and *A. m. carnica* natural populations have been reported to have intermediate hybrid forms with *A. m. ligustica*, derived from the geographic contact of the regions where they are located [8,47,48,49,50]. *A. m. mellifera* natural populations have been originally localized in small areas along the Alpine arch borders, in France and Switzerland, with relevant populations in Liguria. *A. m. carnica* natural populations have been originally identified along the border with Austria and Slovenia, with penetration in the Friuli-Venezia Giulia and Veneto regions [6,25,40,42,47,48,49,50].

Due to recent beekeeping activities and the trade of hybrid queen bees, the original distribution of *A. mellifera* populations and lineages has been modified in Europe, as demonstrated in several European countries, using mitochondrial and nuclear genome markers [1,2,4,5,43]. Since the study of Franck et al. [40], published about 20 years ago, no other studies have investigated the distribution of the main honey bee mtDNA lineages in Italy.

We recently developed a simple end-point PCR method to discriminate the main three mtDNA lineages (A, C and M) of *A. mellifera*, using honey as a source of environmental DNA (eDNA), which also contains traces of the DNA of the honey bees that have produced it [51]. This is a cost-effective method that simplifies the collection of useful specimens, such as the honey, which, however, can be analyzed by investigating small DNA fragments, due to the highly degraded DNA that it contains [52,53,54]. The method gives the possibility to analyze more than one colony at the same time, considering that the honey that is prepared by the beekeepers is usually a mixture obtained from several colonies, or is even derived by different apiaries of the same beekeeper [51,53,54]. Therefore, honey can be used to obtain a quite extensive population-wide picture of the presence of genetic features in a vast geographic area. We already applied this approach to monitor, starting from honey DNA, the diffusion of the honey bee trypanosome parasite *Lotmaria passim* in the north of Italy [55].

In this study, we used the honey samples collected all over Italy as a source of honey bee DNA, to produce an updated distribution map on the main *Apis mellifera* mtDNA lineages in the Italian peninsula, and in the two main Italian islands, Sicily and Sardinia.

## 2. Materials and Methods

### 2.1. Honey Samples

A total of 607 honey samples, produced in the year 2018, were collected. These samples were produced in all 20 Italian regions, by a total of 550 different beekeepers (Table 1). Therefore, the following two lists of honey samples were considered: 550 samples, each obtained from a different beekeeper (indicated as “unique” samples); 58 samples obtained from some of the same beekeepers who provided the “unique” samples. These latter samples were indicated as “redundant” samples even if they were produced from different botanical sources, in different periods of the year and, potentially, only partially from the same apiaries and colonies from which the “unique” samples were obtained. The “redundant” samples were produced by 33 different beekeepers in the Emilia-Romagna (24 beekeepers) and Sardinia (9 beekeepers) regions. These 33 beekeepers provided two to seven different honey samples (Table S1).

### 2.2. DNA Extraction

DNA extraction from honey samples was performed following the protocol previously described and that first included a preparative phase [51]. Starting with the pre-treatment step, 50 g of honey was equally divided into four 50 mL tubes and then diluted with 40 mL of ultrapure water (in each of the four tubes), vortexed and incubated at 40 °C for 30 min. The four tubes for each honey were then centrifuged for 25 min at 5000× *g* at room temperature and the supernatant was discarded. Then, after the resuspension of the pellet with 5 mL of ultrapure water, the rehydrated pellet of the four tubes for each honey sample was merged into one and diluted again with ultrapure water. After a centrifugation step (25 min; 5000× *g*, room temperature) the supernatant was discarded and the pellet was resuspended in 0.5 mL of ultrapure water.

The DNA extraction protocol was based on a CTAB (2% (*w*/*v*) cetyltrimethylammonium bromide; 1.4 M NaCl; 100 mM Tris-HCl; 20 mM EDTA; pH 8) extraction buffer; One milliliter of CTAB extraction buffer and 5 μL of RNase A solution (10 mg/mL) were added to each prepared resuspended pellet. Then, samples were incubated at 60 °C for 10 min and, after this incubation, 30 μL of proteinase K (20 mg/mL) was added before another incubation period at 65 °C for 90 min with gentle mixing. After this step, samples were cooled at room temperature and centrifuged for 10 min at 16,000× *g*. Then 700 μL of the resulting supernatant was transferred in a tube containing 500 μL of chloroform/isoamyl alcohol (24:1), vortexed and centrifuged for 15 min (16,000× *g* at room temperature). The obtained supernatant was transferred in a new 1.5 mL tube for the precipitation of the DNA after the addition of 500 μL of isopropanol and the subsequent washing step with 500 μL of 70% ethanol. Finally, the DNA pellets were rehydrated with 30 μL of sterile water and used immediately or stored at −20 °C until use in PCR analyses.

The quality check of the extracted DNA was performed using the nanophotometer IMPLEN P300 (Implen GmbH, Munchen, Germany) and by running 1% agarose gel electrophoresis in TBE 1X buffer with a staining step with 1X GelRed nucleic acid gel stain (Biotium Inc., Fremont, CA, USA).

### 2.3. PCR and Sequencing Analyses

The primer pair used in this study (E2, forward: 5′-GGCAGAATAAGTGCATTG-3’; reverse: 5′-TTAATATGAATTAAGTGGGRAAW-3′) was reported by Utzeri et al. [51], which includes the reverse primer, newly designed by the authors, and the forward primer (E2), designed by Cornuet et al. [56]. A schematic representation of the amplified mtDNA region with the information on the diagnostic sites of the three main groups of mitotypes (A, C and M), indicated simply as mitotypes thereafter, is reported in Figure S1.

PCR analyses were carried out in a total volume of 14 μL using KAPA HiFi HotStart master mix (Kapa Biosystems, Roche Molecular Systems, Basel, Switzerland) on a 2700 thermal cycler (Life Technologies; Carlsbad, CA, USA). The PCR profile was set following Utzeri et al. [51] and the amplified DNA fragments were electrophoresed in 4.0% agarose gels in TBE 1X buffer and stained with 1X GelRed nucleic acid gel stain (Biotium Inc., Fremont, CA, USA) [51]. Examples of electrophoretic patterns obtained with the fragments of the three main mtDNA lineages are reported in Figure S2. To check the specificity of the amplification, we sequenced 10 amplicons for each lineage, obtained from random samples. Bands selected for the sequencing reactions were purified from the agarose gels and then prepared for Sanger sequencing [51]. Sequencing reactions were loaded on an ABI3100 Avant genetic analyzer sequencer (Applied Biosystems, Foster City, CA, USA) following the chain termination protocol BrightDye^®^ terminator cycle sequencing kit (NIMAGEN, Nijmegen, the Netherlands) provided by the manufacturers. Electropherograms were visually inspected using MEGA X [57] and the online BLASTN tool (http://www.ncbi.nlm.nih.gov/BLAST/ (accessed on 10 May 2021)) was used to compare and validate the attribution of the obtained DNA sequences to the *A. mellifera* mtDNA region of interest.

### 2.4. Data Analyses

Honey samples were positioned on the map of Italy using longitude and latitude coordinates obtained, starting from the localities of the apiaries provided by beekeepers (or using the intermediate locality between more than one apiary positioned in the same municipality or, when the precise apiary information was not available, using the coordinates of the municipality (“Comune”), in which the honey was produced) through a geocoding plugin implemented in QGIS 3.18.0 (QGIS.org, 2021, QGIS Geographic Information System, QGIS Association, http://www.qgis.org (accessed on 5 June 2021). Summary data per region (frequency distribution of honey samples with different mitotype combinations) were calculated. ArcGIS Online (ESRI, https://www.arcgis.com/index.html (accessed on 5 June 2021) was used to create density maps with the Calculate Density tool. Density maps were based on honey samples that contained the targeted mitotypes (alone or in combination with others). Logistic regression was used to test relationships between the distribution of a particular lineage or combination of mitotypes over geographic coordinates. Latitude positions were analysed over the whole Italian peninsula, including or excluding the following two Italian islands: Sardinia and Sicily. Longitude positions were analysed over all regions of the north of Italy (Piedmont, Valle d’Aosta, Liguria, Lombardy, Trentino-Alto Adige, Veneto, Friuli-Venezia Giulia and Emilia-Romagna). In the models, mitotypes were coded as binary variables accounting for honey samples with or without one mitotype (including or excluding honey samples with multiple mitotypes). Fisher exact test (two tailed) was used to compare the frequency of occurred groups of mitotypes or mitotype patterns between groups of honey samples produced by the same beekeepers (“unique” and “redundant” samples).

## 3. Results

### 3.1. Description of the Main Honey-Derived Mitotype Patterns by Italian Region

All of the sequenced fragments of the three mitotypes corresponded to the expected mtDNA regions, as already demonstrated [51]. Table 1 reports the summary of the results, divided by the region of honey production. Figure S3 includes information about the frequency of the honey samples with different mitotype patterns positioned in the administrative map, with the indication of the 20 Italian regions listed in Table 1.

For a total of 410 (“unique”) or 458 (“unique” and “redundant”) honey samples, only one group of mitotypes (C or A) was identified (74.54% or 75.45% of the analyzed two groups of samples, respectively). The honey samples that showed only lineage C were the most frequent ones in all of the regions, except in Sicily (23.08%) and Trentino-Alto Adige (40%). None of the honey samples showed only lineage M. This lineage was always identified in combination with only the A lineage or the C lineage, or with both the A and C lineages (see below). Honey samples that showed only lineage A were very frequent in Sicily (46.15%), as expected, according to the mitotypes reported in the *A. m. siciliana* subspecies [40,51]. Two other honey samples, one produced in Trentino-Alto Adige and one in the Lazio regions, showed only lineage A.

Considering all of the 607 analyzed samples, the frequency of those that reported more than one mitotype was 24.56%, and this ranged from a minimum of 9.09% in Friuli-Venezia Giulia to a maximum of 44.44% in Calabria, with frequencies that exceeded 30% in five other regions (Lombardy, Veneto, Molise, Puglia, and Sicily). The honey samples that showed all three lineages (A + C + M) were produced in 17 out of 20 regions, with the highest frequency being in Puglia (26.47%), Calabria (22.22%), Campania (19.23%), and Lombardy (17.39%). The presence of more than one lineage in the analyzed samples is due to the fact that usually honey derived by one beekeeper is not obtained by just one colony, thus more maternal lineages are possible.

If we consider the number of samples that showed at least the target lineage (for example A, C, or M, whether alone or in combination), lineage C was identified in all of the samples of most of the regions, with the only exception of Trentino-Alto Adige, Lazio, and Sicily. Lineage M was identified in a total of 23.23% of all the samples (with some samples in all 20 regions), ranging from 9.09% in Friuli-Venezia Giulia to 44.44% in Calabria. Lineage A was not identified in three regions only (Valle d’Aosta, Liguria, and Friuli-Venezia Giulia); however, these together accounted for just 6.10% of all of the investigated samples. The highest frequency of samples, including the A mitotypes, was identified in Sicily (61.54%), where, also, the only two samples with the A + M profile were observed.

### 3.2. Comparison of Mitotype Results between Honey Samples Produced by the Same Beekeepers

The “redundant” samples (n = 58), obtained from the beekeepers of the Emilia-Romagna and Sardinia regions, gave the possibility to evaluate if only one sample per beekeeper could be a good approximation of its whole honey bee queen population (Table S1). A total of 19 beekeepers (16 from Emilia-Romagna and three from Sardinia) provided two samples. Of these pairs of samples, 17 were concordant, which means that they had the same mitotype patterns. Only two pairs were not concordant, as in one sample the pattern was only C, whereas the other sample had the C + M pattern in both cases. When the number of samples provided by the beekeepers increased (from three to seven; 14 beekeepers), the concordant/discordant results for all of the samples provided by the same beekeeper were obtained for the following combinations: (1) five of six triplets of samples showed concordant results; (2) four four-samples provided by the same beekeeper, out of five four-samples, had the same mitotype patterns; (3) six samples were provided by only one beekeeper, three of which showed only the C mitotypes and three had the C + M pattern; and (4) seven samples provided by only one beekeeper all showed the same pattern. Summarizing, for six out of 33 cases (18.18%), the results obtained from the honey samples provided by the same beekeeper were not always the same for all of the samples. The frequency of the discordant results was, however, lower for the beekeepers that provided only two samples (10.52%) than for the beekeepers that provided three or more samples (28.57%). However, if we compare, for both regions, the results obtained, including or excluding “redundant” samples, or if we compare “unique” samples versus “redundant” samples, no significant differences in the frequency of mitotypes or mitotype patterns could be observed (*p* > 0.10).

### 3.3. Distribution of Lineages over Geographic Coordinates

**Figure 1:**
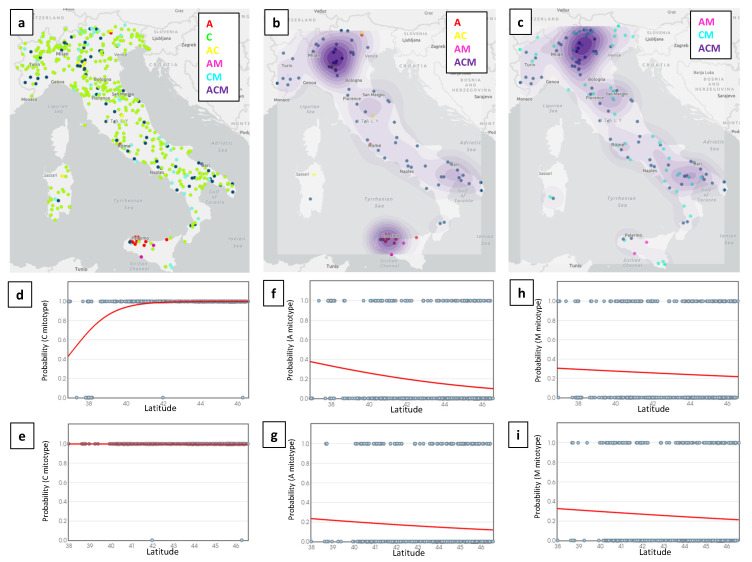
(**a**) Distribution of all analyzed honey samples in Italy with information on the mitotype patterns indicated with dots in the map with the same colors of the legend. (**b**) Density map of the analyzed samples carrying the A lineage. (**c**) Density map of the analyzed samples carrying the M lineage. (**d**–**i**) Logistic regression curves showing relationships between the occurrence of lineage C (**d**,**e**), including all Italian regions, i.e., the peninsula, Sardinia and Sicily (**d**); or only the peninsula data (**e**); lineage A (**f**,**g**), with all Italian regions (**f**); or only the peninsula data (**g**); lineage M (**h**,**i**), with all Italian regions (**h**); or only the peninsula data (**i**).

The geographic localization of “unique” honey samples with the obtained mitotype patterns is shown in Figure 1a. The density maps of honey samples with the A and M lineages (alone or in combination with other groups of mitotypes) are reported in Figure 1b,c, respectively.

Figure S4 reports the density distribution of all the unique samples for comparison with the density maps of the A and M lineages. Lineage A had two major density areas; one was mainly in the north of Italy, centered in the Lombardy region, and one was in Sicily. The two other minor density zones for this lineage, one in Central Italy (centered in the Umbria and Marche regions) and one in the south of Italy (centered in the Puglia and Basilicata regions), could be evidenced (Figure 1b). Lineage M had almost overlapping density areas, excluding the hot spot of Sicily that is highly specific for the A mitotypes. The overlapping picture of the A and M lineage maps is mainly due to the high density of honey samples with the A + C + M mitotype pattern, which indicates regions of potential high mtDNA lineage heterogeneity. Comparing the density map produced with all of the samples (Figure S4) with the two A and M lineage-specific density maps, the hot spot of the north of Italy does not completely match the corresponding sample density, suggesting that an increased frequency of A and M mitotypes is present in the middle of the north of Italy, in the Po valley.

Logistic regression (Table 2 and Table S2) indicated that latitude was a significant predictor of A mitotypes in honey samples, only if Sicily was included in the analysis, together with the data from the peninsula and Sardinia (*p* = 0.0001; Figure 1f), or together only with the data from the peninsula (*p* < 0.0001; Table S2). When Sicily was excluded, the latitude did not explain any distribution profile of the A lineage across the peninsula length (*p* = 0.112; Table 2 and Figure 1g). A similar latitude effect was observed for the C mitotypes only when Sicily was included in the model, mainly due to the high frequency of honey samples carrying the A lineage in this region, which, in turn, reduced the frequency of the samples with the C mitotypes (Table 2 and Table S2, Figure 1d,e). The models that included latitude as predictor of the M lineage in honey samples did not show any significant trend (Table 2 and Table S2; Figure 1h,i). None of the other models involving latitude as predictor, and different mitotype patterns in honey, reported any significant effect (Table 2 and Table S2). When the longitudes of the samples from the regions of the north of Italy were included in the logistic regression models, no significant geographical gradient could be identified for all of the lineages or lineage combinations (Table S2).

## 4. Discussion

In this study, we used an unconventional approach [51] to monitor the distribution of the three main honey bee mtDNA lineages in Italy. The method has some limitations, but also several advantages that should be considered to correctly interpret the results.

Honey DNA is usually highly degraded and only short DNA fragments can be easily amplified by PCR from this template [51,52,53,54,55]; that means that the informativity of the obtained mtDNA amplicons is limited by the sequence information contained in a short fragment, which, in our case, was constituted by a part of the COI-COII intergenic spacer, including the 3′-end of the tRNALeu gene and the non-coding P and Q regions [26,51,56]. As a consequence, it was not possible to obtain a more detailed classification within the main branches of the A, C and M lineages. Another specific characteristic of the applied method is that more than one lineage can be amplified from the same honey sample, as 25% of the investigated unique samples contained two (12%) or three (13%) lineages (Table 1). This is due to the fact that more than one colony contributed to the analyzed samples. However, the information on the number of colonies that contributed to the investigated samples, and thus the heterogeneity or homogeneity of the mtDNA lineages, was not available, because the beekeepers did not apply a specific traceability system for their honey. The only information available was the origin, from an apiary or groups of apiaries. In addition, it is also important to mention that the obtained results are only qualitative and not quantitative; it would be also impossible to know how many honey bees carrying the different mitotypes contributed to the traces left in the honey, and then relate this information to the number of colonies from which the honey bees came from. Despite that, in theory, the relative intensity of the electrophoretic bands separated in the agarose gels could be potentially used to have an approximated and rough estimation of the mtDNA lineage contribution. However, we did not consider this information, as it would not be appropriate (or not possible to evaluate in this context) to establish the needed reference samples for a precise comparison, because the PCR amplification curve and the plateau could be affected by the quality of the extracted honey DNA, which is difficult to control/standardize, and because the simple end-point PCR method that we used cannot be correctly interpreted for relative quantifications, or transformed in a qPCR method. It is also worth mentioning that for most of the samples for which more than one mitotype was detected, limited differences in the intensities of the amplified fragments could be noted. Thus, we could only say that, based on our results, one fourth of all the beekeepers had in their apiaries colonies of more than one mtDNA lineage. This is probably an underestimation of the proportion of beekeepers who have mtDNA heterogeneity in their apiaries, as increasing the number of samples for each beekeeper slightly increases the probability of identifying other mitotypes in addition to the most frequent ones. This is demonstrated with the comparison between the “unique” and “redundant” samples. Despite that, the general overall frequency distribution of the honey samples carrying the different mitotypes or mitotype combinations, including or excluding more samples from the same beekeepers (“unique” or “redundant” samples), did not change substantially. Therefore, it would also be possible to consider that what we obtained could also be considered an approximated indication of the overall frequency distribution of the three main mtDNA lineages over all the Italian colonies, even if the rarer mitotypes were probably overestimated (e.g., A and M, in the peninsula and Sardinia).

All these elements that, on the one hand, are regarded as potential limits, on the other hand can be considered advantages. Large population genetic studies need to analyze a high number of specimens, usually covering large regions, which, in turn, might be very demanding in terms of resources to be dedicated for the sampling and then for obtaining the related molecular data. Using eDNA from honey samples can easily provide a more comprehensive sampling of honey bee DNA, with cost-effective possibilities to retrieve molecular information, as in our case, where mitotype information was easily discriminated. As far as we know, all of the previous studies that monitored *A. mellifera* mtDNA lineage distribution over broad geographical areas analyzed single honey bees, usually collected from different colonies (e.g., [1,2,4,5,43,58,59,60]). This approach gave the possibility to obtain more complete information of the mitotypes, but, on the other hand, could not include, in many cases, a very large number of colonies that would be needed to assure an optimal and detailed territorial representation.

The most recent study that investigated the distribution of *A. mellifera* mtDNA lineages in Italy was published more than 20 years ago by Franck et al. [40], who updated the previous preliminary and partial studies [28,39,42,61,62]. Franck et al. [40] indicated that two mtDNA lineages (C and M) were present in *A. m. ligustica* populations that were sampled in a sparse way over the Italian peninsula, demonstrating the hybrid origin of this subspecies. In this study [40], however, morphometric information on the investigated populations, which would be able to confirm the mtDNA lineage, was not provided. Depending on the population (considering only the 14 continental sites [40]), the frequency of the M mitotypes (mainly M4 in the Northwest Alp borders and M7 in all the other sites) ranged from 0% to about 90%. The highest percentage was in one site in the south of Italy, whereas it was ≥60% in all five of the Northwest sites, and it was well balanced (about 50%) in four other sites (one in Lombardy, north of Italy; one in Umbria, central of Italy; and two in the south of Italy, one in Abruzzo and one in Puglia). C1 was the only mitotype identified in a site of Emilia-Romagna, and the most frequent one in another two sites located in Piedmont and Puglia [40]. Honey bees that were indicated to belong to *A. m. siciliana* and were sampled in Sicily carried only the A mitotypes, which were, however, not identified in any other continental sites [40].

Comparing the overall picture determined by Franck et al. [40] with the mtDNA lineages distribution that we determined, by analyzing 550 honey samples collected all over Italy (Figure 1a), some relevant and substantial differences can be clearly evidenced, despite the methodological differences. In our study, the overall frequency of honey samples with the M lineage (23.2%), which can be considered an overestimation of the frequency of colonies of this group of mitotypes, is much lower than the frequency that could be deduced from the data of Franck et al. [40] (~50%), who reported mtDNA data from individual workers that were collected from a few apiaries within a limited number of continental sites. In addition, none of the honey samples we analyzed had only the M lineage, which is also a clear indication of the general relative low frequency of colonies with this group of mitotypes. In the study of Franck et al. [40], a clear overrepresentation of the M mitotypes was evident in the Northwest Alpine border areas, derived by the contiguity with the *A. m. mellifera* populations. This was not evident from our results, which showed, instead, a general overrepresentation of the C lineage in these areas, even if we could not have samples from the same precise sites of Franck et al. [40]. Again, in our study, the A lineage was not only detected in Sicilian honey, but also in the samples collected all over continental Italy, with a quite high density in the Po valley. It was also surprising that honey samples with only the A lineage were from Trentino-Alto Adige (north of Italy) and Lazio (central of Italy), which probably means that all the colonies of the two beekeepers in these regions had only the A lineage. This is the first study that reported the presence of the African mitotypes in continental Italy. Franck et al. [40] identified the presence of the A lineage only in Sicily, carried by *A. m. siciliana* honey bees, confirming the previous results [28,39]. The honey obtained from *A. m. siciliana* had only the A mitotypes, as we already reported [51]. However, if the honey produced in Sicily is obtained not only from *A. m. siciliana*, other mitotypes are detected, even if the frequency of samples with A mitotypes remains high (Table 1). In the current study, the frequency of samples that did not contain the A mitotypes was 39.5%. Munoz et al. [41] already reported that C and M mitotypes are present in Sicilian colonies, which is in agreement with what we reported here.

If we exclude Sicily, which has a peculiar *A. mellifera* mitotype distribution, there is no clear geographic pattern or gradient for all three mtDNA lineages over the rest of Italy. The diffusion of A mitotypes over the peninsula could be eventually explained by a natural south-to-north movement of the A mitotypes, starting from Sicily, but this hypothesis does not hold according to all logistic models. Therefore, the most plausible explanation of the presence of the African mtDNA lineage all over continental Italy points to human-mediated dispersion, probably derived by the importation of non-native subspecies and/or by the extensive use of hybrid queens of not autochthonous lines, referred to as “Buckfast”. The importation of queens from South America, mainly Argentina, where Africanized bees have been identified [63], could have created a potential route of introgression into the Italian colonies. “Buckfast” bees have been reported to be not very homogeneous at the mtDNA, having, in some cases, A mitotypes [2] that could be spread all over Italy by the use of some lines carrying A mitotypes. Similar explanations of the unexpected occurrence of the A lineage have been proposed by some authors, who reported African mtDNA lineages in other European countries. For example, Oleska et al. [60] recently reported that about 2% of the honey bee colonies in East-Central Europe had A mitotypes.

The presence of some continental hotspots for both A and M mitotypes, mainly in the north of Italy, where a high concentration of beekeeping activities and enterprises are located, further support the hypothesis that beekeeping practices might be the main explanation of the updated A and M lineage map distribution of Italy. The M lineage seems to move in parallel with the A mitotypes, suggesting that despite its original presence in the peninsula, it could be possible that since the study of Franck et al. [40], some changes in its distribution could have occurred, driven by the following same events suggested to explain the presence of the A mitotypes in continental Italy: the import of non-native subspecies and the use of hybrid queens, carrying, in this case, the M mitotypes, with subsequent introgression in native populations, which, in turn, might have modified the original *A. m. ligustica* populations that are known to be present in this area. The putative reduction in the frequency of the mtDNA M lineage in Italy could also be due to not only the introduction of the A lineage in the peninsula, but also to the expansion of the C lineage. This explanation could match the results we obtained for the honey samples in the Northwest borders of Italy, where it seems that the C lineage has substituted, at least in part, the M lineage.

The results at the mtDNA level, however, cannot provide any information on the proportion of introgression at the nuclear genome level. Additional studies are needed to obtain this information in the Italian populations, using whole-genome sequencing data or genotyping a large number of nuclear DNA markers (e.g. [5,29,64,65,66,67,68,69]). It would also be important to monitor the level of adaptation to the continental environment and beekeeping systems, and evaluate the productive and behavioral traits of colonies carrying the introgressed A mitotypes compared to those having other lineages. This is needed, considering that their potential origin from imported South American Africanized honey bees can pose some concerns [70,71,72,73,74], which might also open other questions related to the need to eventually regulate the genetic origin of imported queens, as already pointed out by others [60]. At present, the European Union does not have any restrictions on the importation of honey bee genetic material, apart from the issues related to health aspects [75]. The effect of climate change on the maintenance of mtDNA introgressed lineages over continental Italy is also a topic that needs further investigation [8,76]. This is one of the concerns that has driven the proposition and the implementation of the conservation policies of native honey bee genetic resources [1,2,5,6,7].

## 5. Conclusions

The updated distribution map of honey bee mtDNA lineages that we obtained can be useful to design and then evaluate the potential effectiveness of the conservation policies and actions that are addressed to maintain the diversity and integrity of honey bee genetic resources in Italy.

To improve the usefulness of the honey, as a source of the genetic information of the honey bees that have produced it, we are now working in the following two directions: (1) increasing the geographical density of the honey samples, also saturating some regions that at present are not well covered; (2) producing simple methods that are applicable to honey DNA, to analyze several other honey bee mtDNA diagnostic sites. To obtain a complete overview of the level of introgression that the Italian honey bee populations might have experienced over the last 20 years, it would be important to complement the information we obtained about the distribution of the main mtDNA lineages with nuclear genome data.

## Figures and Tables

**Table 1:** Summary results provided for the 20 Italian regions about the number of honey samples that showed different mitotype patterns and about the frequency of samples carrying at least one of the three main groups of mitotypes (A, C and M). Results have been reported including “unique” and “redundant” samples for the Emilia-Romagna and Sardinia regions.

Regions ^1^	No. of Samples ^2^	No. of Samples with Different Profiles ^2,3^						Frequency of Honey Samples with the Indicated Lineage ^2,4^		
		Only A	Only C	AC	AM	CM	ACM	A	C	M
Piedmont (Piemonte)	56	0	46	0	0	3	7	0.125	1.000	0.179
Valle d’Aosta	8	0	7	0	0	1	0	0.000	1.000	0.125
Liguria	7	0	6	0	0	1	0	0.000	1.000	0.143
Lombardy (Lombardia)	69	0	48	3	0	6	12	0.217	1.000	0.261
Trentino-Alto Adige	20	1	8	0	0	8	3	0.200	0.950	0.550
Veneto	41	0	28	1	0	7	5	0.146	1.000	0.293
Friuli-Venezia Giulia	22	0	20	0	0	2	0	0.000	1.000	0.091
Emilia-Romagna	63 (100)	0 (0)	52 (83)	0 (0)	0 (0)	4 (7)	7 (10)	0.111 (0.110)	1.000 (1.000)	0.175 (0.170)
Tuscany (Toscana)	37	0	29	0	0	4	4	0.108	1.000	0.216
Umbria	15	0	12	1	0	1	1	0.133	1.000	0.133
Marche	20	0	16	0	0	0	4	0.200	1.000	0.200
Lazio	26	1	18	0	0	4	3	0.154	0.962	0.269
Abruzzo	19	0	16	0	0	1	2	0.105	1.000	0.158
Molise	9	0	6	0	0	2	1	0.111	1.000	0.333
Campania	26	0	19	0	0	2	5	0.192	1.000	0.269
Puglia	34	0	23	0	0	2	9	0.265	1.000	0.324
Basilicata	20	0	12	0	0	5	3	0.150	1.000	0.400
Calabria	9	0	5	0	0	2	2	0.222	1.000	0.444
Sicily (Sicilia)	26	12	6	0	2	4	2	0.615	0.462	0.231
Sardinia	23 (43)	0 (0)	19 (36)	1 (1)	0 (0)	2 (3)	1 (3)	0.087 (0.093)	1.000 (1.000)	0.130 (0.140)
Italy	550 (607)	14 (14)	396 (444)	6 (6)	2 (2)	61 (65)	71 (76)	0.169 (0.161)	0.971 (0.974)	0.240 (0.232)

^1^ The 20 Italian regions are listed by the following geographic zones: north, center, south and islands. The Italian name of some regions is provided within brackets. ^2^ The number of the total analyzed samples that included both “unique” and “redundant” samples is reported within brackets. ^3^ Results have been reported including honey samples with the indicated mitotype pattern. ^4^ Frequency of the honey samples that showed the indicated groups of mitotypes, alone or in combination with another one or with the other two lineages.

**Table 2:** Results of the logistic regression models between mitotypes or mitotype patterns in the honey samples and their latitude positions. Dependent variables, constituted by honey samples with different mitotype lineages or mitotype patterns, were coded as binary variables (yes or no).

Mitotypes ^1^	Latitude ^2^	Constant ^3^	Chi-Square ^4^	Odd Ratio (95% CI) ^5^
Only C (P + Sa + Si)	0.130 (0.038); 0.0007	−4.665 (1.657); 0.005	11.398; 0.0007	1.139 (1.056, 1.227)
Only C (P)	0.052 (0.050); 0.302	−1.2179 (2.197); 0.579	1.056; 0.304	1.053 (0.955, 1.162)
C (P + Sa + Si)	0.974 (0.184); <0.0001	−36.189 (7.197); <0.0001	58.965; <0.0001	2.647 (1.845, 3.798)
C (P)	−0.064 (0.370); 0.864	8.318 (16.343); 0.611	0.031; 0.861	0.938 (0.454, 1.938)
A (P + Sa + Si)	−0.176 (0.045); 0.0001	5.979 (1.919); 0.002	15.235; 0.0001	0.839 (0.768, 0.916)
A (P)	−0.096 (0.060); 0.112	2.458 (2.635); 0.351	2.478; 0.116	0.909 (0.807, 1.023)
M (P + Sa + Si)	−0047 (0.040); 0.240	0.913 (1.743); 0.600	1.365; 0.243	0.954 (0.882, 1.032)
M (P)	−0.069 (0.051); 0.176	1.890 (2.226); 0.396	1.812; 0.178	0.934 (0.845,1.031)
ACM (P + Sa + Si)	−0.040 (0.051); 0.432	−0.166 (2.216); 0.940	0.609; 0.435	0.961 (0.869, 1.062)
ACM (P)	−0.127 (0.062); 0.043	3.677 (2.716); 0.176	4.031; 0.447	0.881 (0.780, 0.996)
Multiple mitotypes (P + Sa + Si)	−0.038 (0.040); 0.338	0.578 (1.725); 0.738	0.911; 0.340	0.963 (0.890, 1.041)
Multiple mitotypes (P)	−0.0535 (0.050); 0.289	1.273 (2.207); 0.564	1.118; 0.290	0.948 (0.859,1.046)

^1^ Dependent variables were based on honey samples with the following mitotype lineages or mitotype patterns: only C lineage in the honey, all honey samples including the C lineage, all honey samples including the A lineage, all honey samples including the M lineage, or patterns including all three main groups of mitotypes or more than one group of mitotypes, i.e., multiple mitotypes. The used mitotype information was from honey samples produced in all regions of the Italian peninsula, Sardinia and Sicily (P + Sa + Si) or only in the peninsula (P). ACM indicates honey samples that had the pattern including all three groups of mitotypes. “Multiple mitotypes” indicates honey samples that had the pattern with two or three groups of mitotypes. ^2^ X_1_ variable: values indicate regression coefficients, their standard errors (in brackets) and the *p*-values. ^3^ Constant variable: values indicate regression coefficients, their standard errors (in brackets) and the *p*-values. ^4^ Values of the Chi-square and the probability of the test in the model. ^5^ Odd ratio: the predictor’s effect on the exponential function of the regression coefficient; CI: confidence interval.

## Supplementary Materials

The following are available online at https://www.mdpi.com/article/10.3390/insects12070620/s1, **Figure S1**: Alignment of the targeted *Apis mellifera* mitochondrial DNA (mtDNA) region with the gaps indicated with “-“ that can discriminate the following three main lineages [51]: A (152 bp), M (138 bp) and C (85 bp). Reported sequences are from the A1 mitotype (Gen-Bank/EMBL accession number: EF033649), the M4 mitotype (FJ743637) and C1 mitotype (FJ478010). Other mitotypes of the same lineages might differ in size for a few nucleotides. More details are reported in Utzeri et al. [51], which includes an extended alignment of *A. mellifera* mtDNA sequences. The PCR primer regions are underlined and in bold. **Figure S2:** Examples of gel electrophoresis patterns of the *Apis mellifera* mtDNA amplified fragments obtained from the DNA of the following several honey samples (lanes from 1 to 5): A lineage (band of 152 bp), M lineage (band of 138 bp) and C lineage (band of 85 bp). L: DNA ladder. Some honey samples had more than one band as described in the main text. **Figure S3:** Frequency distribution map, divided by Italian regions, of the analyzed honey samples, which resulted to have the indicated mtDNA lineage patterns (see also Table 1). The size of the pie charts reported for each region is proportional with the total number of analyzed samples produced in the corresponding regions. The colors in the pie charts match with the colors of the patterns indicated in the legend. **Figure S4:** Density map of the unique honey samples analyzed in this study. Dots indicate the geographical localization of the apiaries of each analyzed honey (see Materials and Methods for details). **Table S1:** beekeepers who provided more than one honey sample and the mitotype patterns obtained for each sample. **Table S2:** Results of the logistic regression models (not reported in Table 2) between mitotypes or mitotype patterns in the honey samples and their latitude or longitude positions. Dependent variables (only C mitotypes in the honey, all honey samples including the C mitotypes, all honey samples including the A mitotypes, all honey samples including the M mitotypes or patterns including all three groups of mitotypes or more than one group of mitotypes, i.e., multiple mitotypes) were coded as binary variables (yes or no).
