# Supplementary material for: Distribution of the Main Apis mellifera Mitochondrial DNA Lineages in Italy Assessed Using an Environmental DNA Approach
Source: Insects. 2021 Jul 8;12(7):620. doi: 10.3390/insects12070620 (PMC8304627; doi:10.3390/insects12070620)
Supplement: Supplementary file 1 [file insects-12-00620-s001.zip › insects-1277458-supplementary.pdf]

insects

MDPI

Supplementary Material

Distribution of the main *Apis mellifera* mitochondrial DNA lineages in Italy assessed using an environmental DNA approach

Valerio Joe Utzeri, Anisa Ribani, Valeria Taurisano, Carles Hernández i Banqué and Luca Fontanesi

```
A   GGCAGAATAAGTGCATTGAACTTAAGATTCAAATATAAAGTATTTTTAAACTTTTATTAAAATTAATAAATTAATATAAAATAAAACAAAATATAACAAAATATATTTATTAAAATTTAATTTATTAAAATTCCCCACTTAATTCATATTAA

M   GGCAGAATAAGTGCATTGAACTTAAGATTCAAATATAAAGTATTTTTAAACTTTTATTAAAATTAATAAATTAATATAAAATA------------TAAATTATATTTATTAAAATTTAATTTATTAAAATTTCCCACTTAATTCATATTAA

C   GGCAGAATAAGTGCATTGAACTTAAGATTCAAATATAAAGTATTTTTAAACTTTTATTAAAA-------------------------------------------------------------------TTTCCCCACTTAATTCATATTAA
```

Figure S1. Alignment of the targeted *Apis mellifera* mitochondrial DNA (mtDNA) region with the gaps indicated with "-" that can discriminate three main lineages [51]: A (152 bp), M (138 bp) and C (85 bp). Reported sequences are from the A1 mitotype (GenBank/EMBL accession number: EF033649), the M4 mitotype (FJ743637) and C1 mitotype (FJ478010). Other mitotypes of the same lineages might differ on size for a few nucleotides. More details are reported in Utzeri *et al.* [51], which includes an extended alignment of *A. mellifera* mtDNA sequences. The PCR primer regions are underlined and in bold.

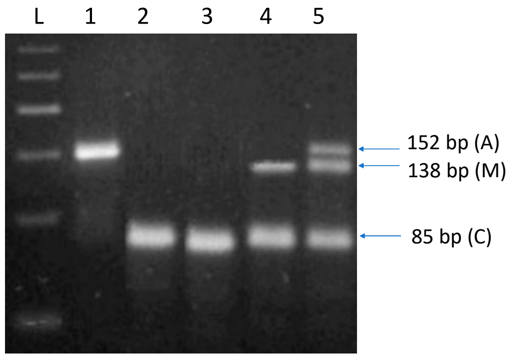

Figure S2. Examples of gel electrophoresis patterns of the *Apis mellifera* mtDNA amplified fragments obtained from the DNA of several honey samples (lanes from 1 to 5): A lineage (band of 152 bp), M lineage (band of 138 bp) and C lineage (band of 85 bp). L: DNA ladder. Some honey samples had more than one band as described in the main text.

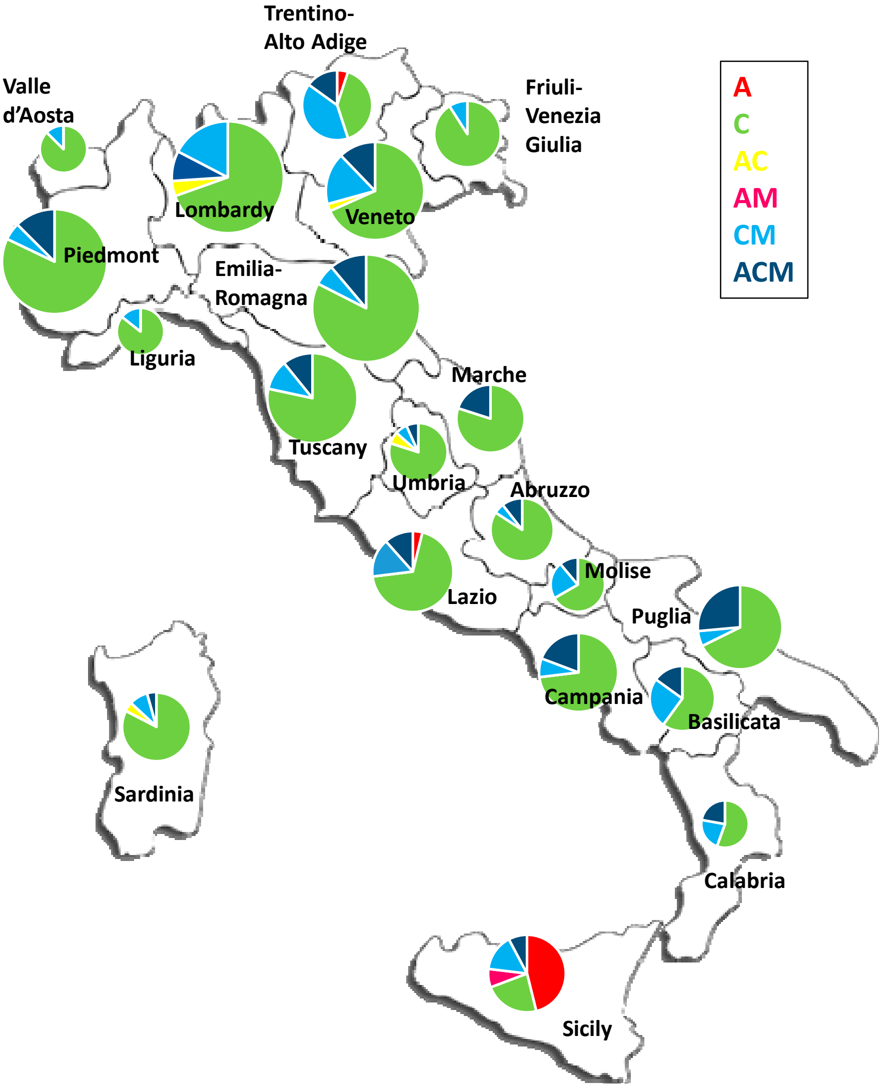

Figure S3. Frequency distribution map, divided by Italian regions, of the analysed honey samples which resulted to have the indicated mtDNA lineage patterns (see also Table 1). The size of the pie charts reported for each region is proportional with the total number of analysed samples produced in the corresponding regions. The colours in the pie charts match with the colours of the patterns indicated in the legend.

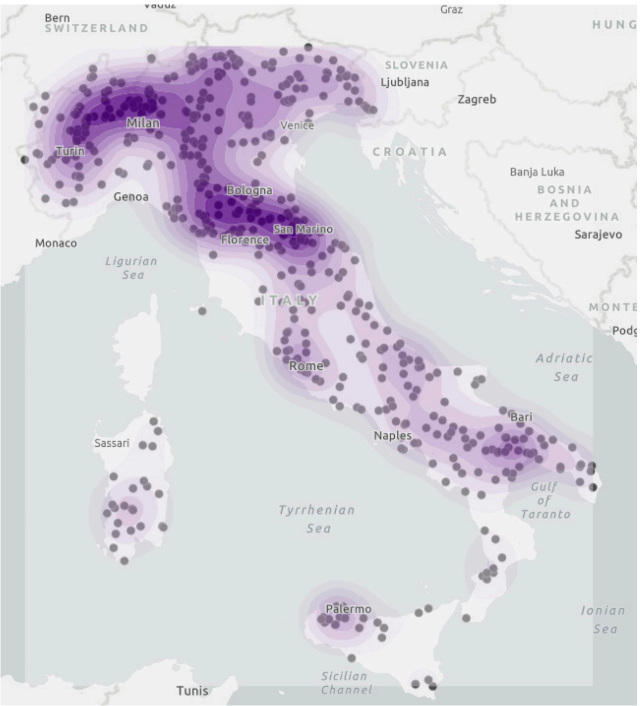

Figure S4. Density map of the unique honey samples analysed in this study. Dots indicate the geographical localisation of the apiaries of each analysed honey (see Materials and Methods for details).

Table S1. Beekeepers who provided more than one honey sample and the mitotype patterns obtained for each sample.

Region	Beekeeper ID	N. of analysed samples	Mitotype patterns [1]
Emilia-Romagna	1	2	C - C
	2	2	C - C
	3	2	C - C
	4	2	C - C
	5	2	C - C
	6	2	C - C
	7	2	C - CM
	8	2	C - C
	9	2	ACM - ACM
	10	2	ACM - ACM
	11	2	C - C
	12	2	C - C
	13	2	C - C
	14	2	C - C
	15	2	CM - C
	16	2	C - C
	17	3	C - C - C
	18	3	C - C - C
	19	3	C - C - C
	20	3	ACM - ACM - C
	21	4	C - C - C - C
	22	4	C - C - C - C
	23	4	C - C - C - C
	24	6	C - C - C - CM - CM - CM
Sardinia	1	2	C - C
	2	2	C - C
	3	2	C - C
	4	3	C - C - C
	5	3	C - C - C
	6	4	ACM - ACM - ACM - C
	7	4	C - C - C - C
	8	5	C - C - C - CM - CM
	9	7	C - C - C - C - C - C- C

[1]The mitotype pattern obtained for each sample is separated with "-". One sample, selected randomly, among the samples provided by the same beekeeper was included in the list of the unique samples. Its mitotype pattern is the fist indicated in the corresponding line.

Table S2. Results of the logistic regression models (not reported in Table 2) between mitotypes or mitotype patterns in the honey samples and their latitude or longitude positions. Dependent variables (only C mitotypes in the honey, all honey samples including the C mitotypes, all honey samples including the A mitotypes, all honey samples including the M mitotypes or patterns including all three groups of mitotypes or more than one group of mitotypes, i.e. multiple mitotypes) were coded as binary variables (yes or no). See also notes to Table 2.

**Mitotypes** [1]	**Lat./Long.**[2]	**Geographic coordinates** [3]	**Constant** [3]	**Chi-Square** [4]	**Odd Ratio (95% CI)** [5]
Only C (P+Si)	Latitude	0.156 (0.040); 0.0001	-5.867 (1.737); 0.0007	15.256; 0.0001	1.169 (1.081, 1.265)
C (P+Si)	Latitude	0.9402 (0.171); <0.0001	-35.050 (6.677); <0.0001	61.567; <0.0001	2.560 (1.833, 3.577)
A (P+Si)	Latitude	-0.204 (0.046); <0.0001	7.250 (1.982); 0.0003	19.341; <0.0001	0.815 (0.745, 0.893)
M (P+Si)	Latitude	-0.068 (0.042); 0.105	1.828 (1.810); 0.312	2.589; 0.108	0.935 (0.862, 1.014)
ACM (P+Si)	Latitude	-0.063 (0.052); 0.228	0.860 (2.268); 0.705	1.417; 0.234	0.939 (0.848, 1.040)
Multiple mitotypes (P+Si)	Latitude	-0.054 (0.041); 0.189	1.304 (1.798); 0.468	1.702; 0.192	0.947 (0.874, 1.027)
Only C	Longitude	-0.036 (0.079); 0.646	1.484 (0.832); 0.074	0.212; 0.645	0.964 (0.826, 1.126)
C	Longitude	-0.581 (0.704); 0.410	12.097 (8.369); 0.148	0.815; 0.367	0.560 (0.141, 2.225)
A	Longitude	-0.094 (0.100); 0.349	-0.892 (1.023); 0.384	0.881; 0.348	0.911 (0.749, 1.108)
M	Longitude	0.020 (0.081); 0.807	-1.408 (0.850); 0.098	0.059; 0.807	1-020 (0.870, 1.196)
ACM	Longitude	-0.137 (0.107); 0.199	-0.614 (1.080); 0.570	1.671; 0.196	0.872 (0.708, 1.074)
Multiple mitotypes	Longitude	0.027 (0.080); 0.732	-1.408 (0.834); 0.091	0.118; 0.732	1.028 (0.879, 1.201)

[1] The used mitotype information was from honey samples produced in all regions of the Italian Peninsula and Sicily (P+Si). ACM indicates honey samples that had the pattern including all three groups of mitotypes. "Multiple mitotypes" indicates honey samples that had the pattern with two or three groups of mitotypes.

[2] Geographic coordinates were latitude or longitude. Longitudinal coordinates were from the regions of the North of Italy (Piedmont, Valle d'Aosta, Liguria, Lombardy, Trentino-Alto Adige, Veneto, Friuli-Venezia Giulia and Emilia-Romagna).

[3] Values indicate regression coefficients, their standard errors (in brackets) and the P-values.

[4] Values of the Chi-square and the probability of the test.

[5] CI: confidence interval.
